# Role of Mesenchymal Stem Cell-Derived Extracellular Vesicles in Epithelial–Mesenchymal Transition

**DOI:** 10.3390/ijms20194813

**Published:** 2019-09-27

**Authors:** Sevindzh Kletukhina, Olga Neustroeva, Victoria James, Albert Rizvanov, Marina Gomzikova

**Affiliations:** 1 OpenLab Gene and Cell Technologies, Institute of Fundamental Medicine and Biology, Kazan Federal University, Kazan 420008, Russia; sevindzh.rasulova.1993@mail.ru (S.K.); neustroeva.olga@mail.ru (O.N.); 2 School of Veterinary Medicine and Science, University of Nottingham, Nottingham LE12 5RD, UK; Victoria.James@nottingham.ac.uk; 3 M.M. Shemyakin–Yu.A. Ovchinnikov Institute of Bioorganic Chemistry of the Russian Academy of Sciences, Moscow 117997, Russia

**Keywords:** extracellular vesicles, epithelial–mesenchymal transition, cancer stem cells, intercellular communication

## Abstract

Epithelial–mesenchymal transition (EMT) is a process that takes place during embryonic development, wound healing, and under some pathological processes, including fibrosis and tumor progression. The molecular changes occurring within epithelial cells during transformation to a mesenchymal phenotype have been well studied. However, to date, the mechanism of EMT induction remains to be fully elucidated. Recent findings in the field of intercellular communication have shed new light on this process and indicate the need for further studies into this important mechanism. New evidence supports the hypothesis that intercellular communication between mesenchymal stroma/stem cells (MSCs) and resident epithelial cells plays an important role in EMT induction. Besides direct interactions between cells, indirect paracrine interactions by soluble factors and extracellular vesicles also occur. Extracellular vesicles (EVs) are important mediators of intercellular communication, through the transfer of biologically active molecules, genetic material (mRNA, microRNA, siRNA, DNA), and EMT inducers to the target cells, which are capable of reprogramming recipient cells. In this review, we discuss the role of intercellular communication by EVs to induce EMT and the acquisition of stemness properties by normal and tumor epithelial cells.

## 1. Introduction

Extracellular vesicles (EVs) are membrane-surrounded structures that act as paracrine effectors, as they are released by cells to deliver signals to other cells. EVs can be characterized by size and the mechanism of their biogenesis. The majority of studies investigating intercellular communication focus on an admixture of plasma membrane-released microvesicles (microparticles) and endosome-derived exosomes. Exosomes have sizes ranging from 40 to 150 nm [1,2], microvesicles have a heterogeneous size: from 40 to 2000 nm [3,4]. They impact a variety of biological processes, transferring biologically active molecules and are secreted by virtually all cells of the body [5].

EVs contain in their composition mRNA, microRNA, various proteins, and lipids, which they deliver to neighboring cells and systemically (via the blood and lymphatic system) [6,7]. EVs mediate the connection between cells of the body through receptor–ligand-mediated interactions and/or direct fusion with target cells. Mesenchymal stem cell (MSC)-derived EVs have been shown to alter the phenotype of target cells and modulate the microenvironment [8]. Furthermore, it was shown that EVs derived from embryonic stem cells mediate a horizontal transfer of bioactive molecules and genetic material (including mRNA [9] and microRNA [10]) leading to the reprogramming of target cells.

Epithelial–mesenchymal transition (EMT) occurs in embryonic development, in the adult it can be observed during wound healing, regeneration, organ fibrosis, and cancer [11,12,13,14]. However, the specific stimulus and mechanism of EMT induction in epithelial cells is still unknown. It is known that inflamed tissue and the tumor microenvironment contains a range of cell populations, which include immune cells, endothelial cells, fibroblasts, and mesenchymal stroma/stem cells [15]. We believe that intercellular communication between stem, stromal cells (tissue-specific progenitor cells, mesenchymal stem cells), and epithelial cells contribute to the induction of EMT reprogramming and the acquisition of a MSC phenotype.

## 2. Epithelial–Mesenchymal Transition

The architecture of epithelial cells is usually in the form of a sheet, with tight connections via surface adhesion proteins and apical–basal polarity [16,17]. The key events during EMT are the loss of this apical–basal polarity, cell–cell junctions, and adherence to the basement membrane by epithelial cells, and the appearance of mesenchymal cell properties [17]. The epithelial cells show a reduction in proteins related to apical dense compounds, such as occlusion, claudins, desmoplakin, and plakophilin, as well as inhibition of E-cadherin expression during the EMT [18,19]. In contrast, the epithelial cells increase expression of vimentin and N-cadherin and show traits characteristic of mesenchymal cells including migration and invasion [13], supporting their ability to develop into specific tissues and organs to mediate repair and regeneration [20,21].

Induction of EMT is regulated at the molecular level by a variety of growth factor signals, in particular transforming growth factor-β (TGF-β), hepatocyte growth factor (HGF), epidermal growth factor (EGF), fibroblast growth factor (FGF), Wnt proteins, IL-6, and hypoxia-inducible factor (HIF)-1α [22,23,24,25,26,27,28]. These initial signals lead to changes in gene expression mediated by factors such as Twist1, Twist2, Snail, Zeb1, Zeb2, Slug, which consequently result in mesenchymal-like changes within the epithelial cells [29]. For example, TGF-β is a multifunctional cytokine that is considered the main inducer of EMT. The TGF-β signaling pathway plays an important role in the regulation of cell proliferation, differentiation, invasion, migration, apoptosis, and modification of the microenvironment and also stimulates pathophysiological EMT and metastasis [22,23,24]. HGF activates the c-Met signaling pathway, together they increase invasive and metastatic potential and ensure the survival of cancer cells in the bloodstream in the absence of cell-to-cell contact [26]. EGF and Snail2 play an important role in wound healing, during which signaling through the EGF receptor predominantly activates extracellular signal-regulated kinase (ERK) pathways. It was found that the Erk5 pathway specifically enhances Snail2 promoter activity and controls wound healing in vitro [25]. Other researchers have shown that EGF-stimulated Smad2/3 activation promotes EMT, activates several key EMT markers, inhibits E-cadherin expression, and improves invasion and migration in breast cancer cells [27]. FGF is involved in the formation of the mesenchyme and during EMT in the adult it acts similarly to increase the expression of vimentin and fibroblast-specific protein 1 (FSP1), as well as inducing matrix metallopeptidase 2 (MMP-2) activity increasing cell mobility. FGF is also capable of causing changes in the actin cytoskeleton capable of enabling anchorage-independent growth [28].

EMTs occur in three distinct biological processes. The first type of EMT is associated with implantation, embryo formation, organ development, and formation of a variety of cell types with mesenchymal phenotypes. This type of EMT generates mesenchymal cells (primary mesenchyme), which can later undergo mesenchymal–epithelial transition (MET) with formation of secondary epithelium [30]. EMT type 1 is detected during the formation of the neural crest, gastrulation in the primitive deforming somatic recession, the formation of the heart valve, and other embryological phenomena [31].

EMT type 2 is associated with wound healing, tissue regeneration, and organ fibrosis. This type of EMT is induced by repair-related events that usually generate fibroblasts and other related cells, in order to repair the tissues after trauma and inflammation [32]. During wound healing at the edges of the damaged zone, cells that have undergone EMT are found [33]. For example, keratinocytes located in the border of a wound acquire an intermediate phenotype, which is called a “metastable” state, and gain the ability to move around. In addition, they have markers that are characteristic of MSCs, while in the deeper layers of the skin, this phenomenon is not observed [33].

EMT type 3 occurs in neoplastic cells that have undergone genetic or epigenetic changes. Carcinoma cells that have undergone EMT are able to metastasize, thereby inducing the progression of cancer. Cancer cells may undergo EMT to differing extents, retaining epithelial features and acquiring some mesenchymal traits, or losing all epithelial features and acquiring completely mesenchymal properties. The rate of proliferation, metastasis, likelihood of relapse, and individual response to chemotherapy are all influenced by the switching between MET and EMT states [34]. Recovery of epithelial features allows the proliferation of tumor cells in these secondary tumor clusters in metastases [35]. Stress (toxic, infectious, hemodynamic, metabolic, hypoxia, nutrient deprivation, and inflammation) promotes tumor cells to secrete a spectrum of cytokines and chemokines that favor EMT [36]. In parallel, MSCs are recruited to inflammation sites by chemotaxis [37], communication between tumor cells and MSCs within the tumor microenvironment may impact on EMT and is currently under study.

## 3. Extracellular Vesicles of Stem Cells

The composition of EVs depends on the type of parental cell and conditions under which vesicle release occurs [38] and often reflects the specific expression profile and changes in epigenetic regulation of the EV-producing cell [39,40,41].

Previous studies have shown that MSCs do not require direct contact with neighboring cells to induce regeneration [42,43,44]. In this regard, the stem cell paracrine hypothesis was developed which postulates the paracrine action of transplanted stem cells on target cell by secreting soluble and insoluble factors into the extracellular space [45,46,47,48]. Growing evidence points that EVs possess biological activity similar to that of the parental cell. For example, platelet-derived EVs transfer coagulation factors and participate in blood clotting [49], embryonic stem cell-derived EVs increase survival and improve expansion of recipient cells [9,41,50], cancer cell-derived EVs contain oncogenic molecules and contribute to the remodeling of tumor microenvironment [51]. MSC-derived EVs are of particular interest due to their ability to stimulate regeneration and induce angiogenesis [52,53,54,55].

Cells within a damage site secrete huge amounts of cytokines which attract MSCs toward these areas of inflammation [56,57]. Previous studies have shown that MSC-derived EVs demonstrate similar therapeutic effects to parental MSCs by delivery of biological active molecules to target cells. For example, human embryonic MSC-derived EVs promote osteochondral repair [58]. Human fetal MSC EVs promote liver regeneration by activation the IL-6/STAT3 signaling pathway and cell cycle progression in hepatocytes after CCl4-induced injury in rats [59]. The protective effect of EVs derived from human umbilical cord MSCs was observed using a cisplatin-induced rat nephrotoxicity model [60]. These and other studies have placed MSC-derived EVs as therapeutic tools with significant potential in regenerative medicine.

It is known, that on the surface of EVs is present phosphotidylserine (PS) [61]. Due to the presence of PS and tissue factor in them, EVs have procoagulant and prothrombic properties [62,63]. They also regulate wound healing, inflammation, and vascular integrity [64,65]. Since the tumor niche resembles the site of chronic wound healing [66], and viable cancer cells have high PS levels on the outer surface and exhibit a wide range of surface PSs [67], EVs could be used as a therapeutic tool for treating/detecting a tumor.

Beside protein and lipids, stem cell-derived EVs contain genetic material (mRNA, microRNA, siRNA, DNA) [50,68] and are able to reprogram target cells by horizontal transfer of mRNA [9]. Ratajczak et al. showed that embryonic stem cell-derived EVs increase the pluripotency of target cells by inducing the expression of early pluripotent (Oct-4, Nanog, and Rex-1) and early hematopoietic stem cell (Scl, HoxB4, and GATA 2) markers [9]. MSC-derived EVs deliver RNA into injured tubular cells, altering their gene expression, and inducing dedifferentiation [69]. The accumulating data indicates that stem cell-derived EVs carry biologically active molecules, including transcription factors, and other genetic material capable of inducing viability and reprogramming of target cells toward a stem cell phenotype.

## 4. Migration of MSCs toward Injury, Inflammation Site, and Cancerous Tissues

MSC migrate in response to chemotaxic factors including inflammatory cytokines, growth factors, and chemokines produced by the injured tissue [70]. In fact, it was shown that adipose tissue-derived mesenchymal stem cells (AD-MSCs) and bone marrow-MSCs (BM-MSCs) show enhanced migration capacity toward chemokines and growth factors: platelet-derived growth factor-AB (PDGF-AB), insulin-like growth factor-1 (IGF-1), stromal-derived factor-1 (SDF-1), macrophage-derived chemokine (MDC), TGF-β1, and tumor necrosis factor alpha (TNF-α)—the most active MSC’s chemoattractant [71,72,73]. It is known that inflammation is the reason MSCs migrate from adipose tissue and bone marrow (stem cells niches) to blood and lymph nodes by CXCL12 (SDF-1)/CXCR4-dependent mechanisms [72].

The phenomenon of MSC mobilization in injury sites is utilized in the treatment of many diseases. In inflammation areas, MSCs show beneficial effects on neighboring cells via paracrine stimulation improving cell expansion and survival and preventing apoptosis. For instance, intravenous injection of human MSCs increases corneal allograft engraftment and prevents their rejection through the secretion of anti-inflammatory molecule TNFα-stimulated gene (TSG) 6 [74]. BM-MSCs contribute to reducing wound size by paracrine effects on angiogenesis and fibroblast migration [75]. Nakanishi et al. showed that conditioned medium (CM) taken from MSCs promotes cardiac progenitor cell proliferation and inhibits their apoptosis [76]. In addition, Linero and Chaparro demonstrated that MSC CM promoted bone callus formation [77]. These studies demonstrated that paracrine stimulation of neighboring cells is responsible for the beneficial effects of MSC therapy.

**Figure 1:**
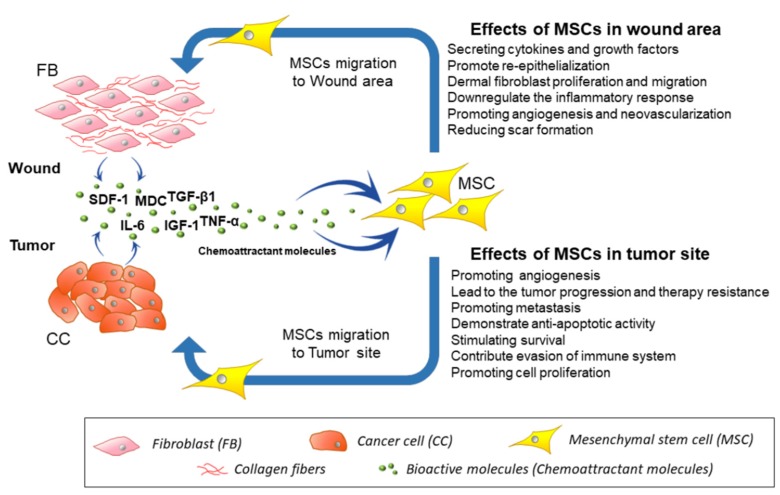
Mesenchymal stem cell (MSC) migration toward tumor/injury site. Tumors or inflamed tissues release the chemokines and growth factors inducing MSC chemotaxis. Migrated MSCs demonstrate beneficial effects on resident cells [37,70,71,72,73,74,75,76,77,79,81,82,87,88,89,90,91,92,93,94,95].

As a tumor is often described as being like a “chronic wound” [78,79,80], the hypoxic conditions and high concentration of cytokines and growth factors IL-1α, IL-1β, IL-6, FGF-2, IGF-1, TGF-β, VEGF-A, HIF-1α, EGF, TGF-α are a likely trigger for MSCs recruitment to tumor microenvironments [79,81,82]. These same factors, which act to control wound healing, are critical not only in MSC recruitment but also in their function. For example, TGF-β1 expressed by prostate cancer cells mediates MSC transdifferentiation into tumor-supporting carcinoma-associated fibroblasts (CAFs) [83,84,85]. Other studies demonstrate some MSCs integrate in a tumor and subsequently transform into tumor-associated MSCs (TA-MSCs). TA-MSCs show a stronger tumor-promoting capacity through microenvironment modulation [86]. MSCs migrate to the tumor microenvironment in much the same way as observed at sites of inflammation and in both cases, they effect to resident cells via paracrine stimulation [37,70,71,72,73,74,75,76,77,79,81,82,87,88,89,90,91,92,93,94,95] (Figure 1). MSC tropism to tumors has been utilized as a means of delivering an antitumor therapy. Ren et al. showed that i.v. injection of MSC-overexpressing lipocalin-2 led to the significant reduction of tumor volume in a model of lung cancer [96]. Intranasal injection of MSCs was found to have strong tropism to gliomas and potentially could be used to target brain tumors [97]. The potential of MSCs to deliver drugs has been demonstrated in a number of tumor types. Systemic delivery of MSC-overexpressing IL-12 significantly inhibited the growth of established subcutaneous renal tumors [98]. Furthermore, modification of MSCs to express TRAIL led to long-term remission of renal cell carcinoma [99].

Tumor-infiltrating MSCs (TA-MSCs) are thought to be important contributors to EMT via direct cell–cell contact and through paracrine mechanisms such as the secretion of bioactive molecules and extracellular vesicles EVs [15].

## 5. Mesenchymal Stem Cells in Epithelial–Mesenchymal Transition and Related Processes

Ectopic expression of EMT transcription factors (TFs), including Twist1 and Snail1 was conducted in order to assess the contribution of EMT to the process of metastasis. It was observed that expression of Twist1 is associated with the acquisition of stemness properties by tumor cells and increased metastasis [100,101]. However these factors did not contribute to the metastatic progression [102,103]. Constitutive expression of EMT-TFs induces a permanent state of EMT and blocks the proposed MET, which is necessary for the development of metastatic sites [100]. Indeed, suppression of Snail1, Twist1, or Prrx1 attenuates the EMT and promotes the colonization of the metastatic site by tumor-initiating cells [102,104,105].

The molecular changes occurring during EMT have been well studied. However, the driver of EMT remains unclear. The presence of MSCs in the tumor stroma is able to stimulate EMT of cancer cells. In models of breast cancer, bone marrow-derived MSCs promote de novo lysyl oxidase (LOX) production from breast tumor cells, this in turn stimulates Twist transcription and triggers EMT. The acquisition of stemness properties through Twist-induced EMT results in increased metastasis to both the lungs and bones [106]. Similarly, TGF-β1 secreted by human AD-MSCs has been shown to regulate EMT in MCF7 (breast cancer cells) by targeting the ZEB/miR-200 regulatory loop [107]. Research has shown that direct co-culture of breast or gastric cancer cells with human BM-MSCs resulted in the upregulation of EMT markers N-cadherin, vimentin, Twist, and Snail and the downregulation of E-cadherin [108,109]. The authors showed that the factors secreted by MSCs are responsible for changes in epithelial/mesenchymal cell markers, the morphology and growth pattern of breast cancer cells, the increased expression of genes associated with invasion and migration, angiogenesis, and anti-apoptosis [108]. MSC supernatant has been used to induce EMT in human hepatocellular carcinoma cells (HCC). HCC cells were grown in CM from MSCs pretreated with TNF-α and IFN-γ. HCC cells showed marked changes in molecular markers and functional characteristics associated with EMT, such as increased migration and invasion in both in vitro and in vivo [110]. CM of spheroid MSC cultures has also been used to demonstrate the effects of MSC-secreted factors. Klopp et al. treated human mammary epithelial cells (HMECs), MCF-7 and SUM149 (breast cancer cells), with MSC-CM. This led to an increase in the formation of the mammosphere by 6.4–21 times. The mammospheres had lower levels of cell adhesion protein, E-cadherin, increased expression of N-cadherin, vimentin, Snail, and Slug, all characteristic of a pro-invasive mesenchymal phenotype [111]. A previous study demonstrated that the CM of human MSCs (hMSCs) promoted the proliferation, migration, and invasion of PC-3 (prostate cancer cells) by upregulating of the expression of matrix metallopeptidase 2 (MMP-2) and matrix metallopeptidase 9 (MMP-9). Blocking TGF-β blunted the pro-oncogenic function of hMSCs. These results suggest that hMSCs play a pro-oncogenic role in the growth of human prostate cancer by producing TGF-β [112].

**Figure 2:**
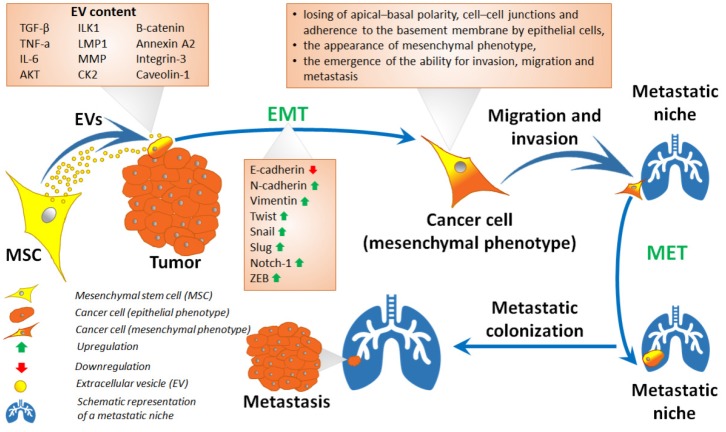
Schematic representation of the epithelial–mesenchymal transition (EMT) mechanism. Tumor cell undergo EMT under the influence of MSC-derived extracellular vesicles (EVs), with subsequent migration to a metastatic niche, returning to the epithelial phenotype (mesenchymal–epithelial transition (MET)) and the formation of a new metastatic site.

Cell-to-cell contact and factors secreted by MSCs have a significant effect on tumor cells and mediating EMT. EVs form an important element of the factors secreted by MSCs. These unique mediators of MSC cell-to-cell communication contain inducers of EMT, such as TGF-β, TNF-a, IL-6, TSG101, RAC-alpha serine/threonine-protein kinase (AKT), integrin-linked kinase (ILK) 1, b-catenin, casein kinase II (CK2), annexin A2, integrin-3, caveolin-1, and matrix metalloproteinases [113,114,115,116,117,118,119,120]. Luga et al. found that EVs produced by breast cancer-associated fibroblasts in the tumor microenvironment contain WNT signaling pathway proteins and are able to activate the migration and metastasis of breast tumor cells [121]. Aga et al. demonstrated that EVs carrying latent membrane protein 1 (LMP1) modulate the expression of EMT markers in recipient cells (N-cadherin expression is increased and E-cadherin is decreased) [122] (Figure 2). Whilst Zhou et al. showed EVs produced by human umbilical cord MSCs significantly enhance the proliferative, migratory, and invasive properties of breast cancer cells (MDA-MB-231 and MCF-7) through the induction of EMT via the ERK pathway in vitro [123].

In metastatic sites, a reversal to a more epithelial state, mesenchymal–epithelial transition, is usually necessary [124]. As with EMT, the drivers of MET are yet to be fully determined [125]. Potentially the loss of secreted factors such as EVs from TA-MSCs may be one drivers of the reversal MET process, as once the cancer cells have migrated from the primary tumor, they no longer face the signals they experienced within the primary tumor microenvironment.

The understanding of the trigger mechanism of EMT of cancer cells is essential for the successful antitumor therapy. To prevent tumor dissemination, drugs and strategies targeted on the EMT program might be developed. Thus, the possible use of natural or modified/loaded EV MSCs to inhibit factors inducing EMT will provide an opportunity to control the process of tumor metastasis For example, in the future, in EVs, you can try to download the compounds: apigenin [126], melatonin [127], miR-711 [127], which have already shown the ability to inhibit migration and invasion, as well as the emergence of EMT of tumor cells. Moreover, to increase the efficiency of the delivery of EV contents, one can use targeting with recombinant proteins without affecting the integrity of the EVs, as was shown in the article by Kooijmans et al. [38].

## 6. Conclusions

Current evidence clearly supports a role for MSCs in the EMT process via both direct cell contact and through secreted factors. However, the full extent of MSCs and their paracrine effects, particularly via EVs, in the process of driving EMT and MET requires further scrutiny. A thorough study of the components of the molecular composition of EVs will help determine which of them contribute most to the induction and progression of EMT and MET. This is an important area of research since the potential of utilizing MSC EVs to manipulate EMT/MET transitions offers a potentially significant therapeutic opportunity to target both metastatic cancer and other chronic inflammatory diseases including chronic, non-healing wounds.
